# Supplementary material for: Resolving the full spectrum of human genome variation using Linked-Reads
Source: Genome Res. 2019 Apr;29(4):635–45. doi: 10.1101/gr.234443.118 (PMC6442396; doi:10.1101/gr.234443.118)
Supplement: Supplemental Material [file supp_gr.234443.118_Supplemental_longranger-2.2.zip › longranger-2.2.tar.bz2!/lr2.2-public/lib/stan/cmdstan/stan/doc/api/html/struct_eigen_1_1internal_1_1general__matrix__matrix__product_3_01_index_00_01stan_1_1agrad_1_1va9b485de9571b6e46d7a64aff43a6d9d3.html]

Stan: Eigen::internal::general\_matrix\_matrix\_product< Index, stan::agrad::var, LhsStorageOrder, ConjugateLhs, stan::agrad::var, RhsStorageOrder, ConjugateRhs, ColMajor > Struct Template Reference


|  |  |
| --- | --- |
|  | Stan  2.5.0  probability, sampling & optimization |


- Main Page
- Related Pages
- Namespaces
- Classes
- Files

- Class List
- Class Index
- Class Hierarchy
- Class Members

All Classes Namespaces Files Functions Variables Typedefs Enumerations Enumerator Friends Macros Pages

- Eigen
- internal
- general\_matrix\_matrix\_product< Index, stan::agrad::var, LhsStorageOrder, ConjugateLhs, stan::agrad::var, RhsStorageOrder, ConjugateRhs, ColMajor >

Public Types |
Static Public Member Functions |
List of all members

Eigen::internal::general\_matrix\_matrix\_product< Index, stan::agrad::var, LhsStorageOrder, ConjugateLhs, stan::agrad::var, RhsStorageOrder, ConjugateRhs, ColMajor > Struct Template Reference

`#include <Eigen_NumTraits.hpp>`

|  |  |
| --- | --- |
| Public Types | |
| typedef stan::agrad::var | LhsScalar |
|  | |
| typedef stan::agrad::var | RhsScalar |
|  | |
| typedef scalar\_product\_traits  < LhsScalar, RhsScalar >  ::ReturnType | ResScalar |
|  | |

|  |  |
| --- | --- |
| Static Public Member Functions | |
| static void | run (Index rows, Index cols, Index depth, const LhsScalar \*\_lhs, Index lhsStride, const RhsScalar \*\_rhs, Index rhsStride, ResScalar \*res, Index resStride, const ResScalar &alpha, level3\_blocking< LhsScalar, RhsScalar > &, GemmParallelInfo< Index > \*) |
|  | |

## Detailed Description

### template<typename Index, int LhsStorageOrder, bool ConjugateLhs, int RhsStorageOrder, bool ConjugateRhs> struct Eigen::internal::general\_matrix\_matrix\_product< Index, stan::agrad::var, LhsStorageOrder, ConjugateLhs, stan::agrad::var, RhsStorageOrder, ConjugateRhs, ColMajor >

Definition at line 216 of file Eigen\_NumTraits.hpp.

## Member Typedef Documentation

template<typename Index , int LhsStorageOrder, bool ConjugateLhs, int RhsStorageOrder, bool ConjugateRhs>

typedef stan::agrad::var Eigen::internal::general\_matrix\_matrix\_product< Index, stan::agrad::var, LhsStorageOrder, ConjugateLhs, stan::agrad::var, RhsStorageOrder, ConjugateRhs, ColMajor >::LhsScalar

Definition at line 218 of file Eigen\_NumTraits.hpp.

template<typename Index , int LhsStorageOrder, bool ConjugateLhs, int RhsStorageOrder, bool ConjugateRhs>

typedef scalar\_product\_traits<LhsScalar, RhsScalar>::ReturnType Eigen::internal::general\_matrix\_matrix\_product< Index, stan::agrad::var, LhsStorageOrder, ConjugateLhs, stan::agrad::var, RhsStorageOrder, ConjugateRhs, ColMajor >::ResScalar

Definition at line 220 of file Eigen\_NumTraits.hpp.

template<typename Index , int LhsStorageOrder, bool ConjugateLhs, int RhsStorageOrder, bool ConjugateRhs>

typedef stan::agrad::var Eigen::internal::general\_matrix\_matrix\_product< Index, stan::agrad::var, LhsStorageOrder, ConjugateLhs, stan::agrad::var, RhsStorageOrder, ConjugateRhs, ColMajor >::RhsScalar

Definition at line 219 of file Eigen\_NumTraits.hpp.

## Member Function Documentation

template<typename Index , int LhsStorageOrder, bool ConjugateLhs, int RhsStorageOrder, bool ConjugateRhs>

|  |  |  |  |
| --- | --- | --- | --- |
| static void Eigen::internal::general\_matrix\_matrix\_product< Index, stan::agrad::var, LhsStorageOrder, ConjugateLhs, stan::agrad::var, RhsStorageOrder, ConjugateRhs, ColMajor >::run | ( | Index | *rows*, |
|  |  | Index | *cols*, |
|  |  | Index | *depth*, |
|  |  | const LhsScalar \* | *\_lhs*, |
|  |  | Index | *lhsStride*, |
|  |  | const RhsScalar \* | *\_rhs*, |
|  |  | Index | *rhsStride*, |
|  |  | ResScalar \* | *res*, |
|  |  | Index | *resStride*, |
|  |  | const ResScalar & | *alpha*, |
|  |  | level3\_blocking< LhsScalar, RhsScalar > & | , |
|  |  | GemmParallelInfo< Index > \* |  |
|  | ) |  |  |

inlinestatic

Definition at line 221 of file Eigen\_NumTraits.hpp.

---

The documentation for this struct was generated from the following file:

- src/stan/agrad/rev/matrix/Eigen\_NumTraits.hpp


---

[ Stan Home Page ]

*© 2011–2014,
Stan Development Team.*
